# Supplementary material for: Staphylococcus aureus-Induced Degeneration of Nociceptive Neurons in Caenorhabditis elegans
Source: bioRxiv. 2025 May 7:2025.05.01.651706. Preprint. [Version 1] doi: 10.1101/2025.05.01.651706 (PMC12247897; doi:10.1101/2025.05.01.651706)
Supplement: Supplement 3 [file NIHPP2025.05.01.651706v1-supplement-3.pdf]

bioRxiv preprint doi: https://doi.org/10.1101/2025.05.01.651706; this version posted May 7, 2025. The copyright holder for this preprint (which was not certified by peer review) is the author/funder, who has granted bioRxiv a license to display the preprint in perpetuity. It is made available under aCC-BY 4.0 International license.

Supporting Information

Supplemental Table 1: *C. elegans* Strains Used

Strain Name	Genotype	Procured From	Figure in this Paper
N2	Bristol Wildtype isolate	CGC	7
CX10536	*kyEx2995[str-2p::GCaMP2.2b, unc-122::GFP]*	CGC	4F, G
CX10979	*N2;kyEx2865[sra-6p::GCaMP3]*	Bargmann Lab	1B-D, 2, 3, Sup 1, Sup 2B-C
JIN2164	*kyEx2865[sra-6p::GCaMP3]; asp-4(ok2693)X*	This work	6B
JIN2166	CX10979;MT4770 *kyEx2865[sra-6p::GCaMP3]; ced-9(n1950)I*	Irazoqui Lab	6A
JIN2167	*kyEx2865[sra-6p::GCaMP3]; hlh-30(tm1978)IV*	This work	6D
JIN2191	*oyIs14[sra-6p::GFP + lin-15(+)]V.; otIs38 [unc-47(del)p::GFP]X*	CGC	4A-C, 5, Sup 3
JIN2192	*oyIs14[sra-6p::GFP + lin-15(+)]V; otIs38[unc-47(del)::GFP]X*	Irazoqui Lab	6
JIN2231	*itr-1(sa73)IV; oyIs14[sra-6p::GFP + lin-15(+)]V; otIs38[unc-47(del)::GFP]X*	This work	6C
JIN2233	*ced-3(n717)IV; oyIs14[sra-6p::GFP + lin-15(+)]V; otIs38[unc-47(del)::GFP]X*	This work	6A
JIN2235	*fat-3(wa22)IV; oyIs14[sra-6p::GFP + lin-15(+)]V; otIs38[unc-47(del)::GFP]X*	This work	6C
JIN2276	*atg-4.2(ola316)IV; oyIs14[sra-6p::GFP + lin-15(+)]V; otIs38[unc-47(del)::GFP]X*	This work	6E
JIN2289	*atg-18(gk378)V ; oyIs14[sra-6p::GFP + lin-15(+)]V; otIs38[unc-47(del)::GFP]X*	This work	6E
N2	Bristol Wildtype isolate	CGC	7
OH14884	*pha-1(e2123) III; him-5(e1490) V; otIs646*	CGC	4D, E

bioRxiv preprint doi: https://doi.org/10.1101/2025.05.01.651706; this version posted May 7, 2025. The copyright holder for this preprint (which was not certified by peer review) is the author/funder, who has granted bioRxiv a license to display the preprint in perpetuity. It is made available under aCC-BY 4.0 International license.

Supplemental Video 1: *C. elegans* ASH neuron after 3 hours of *E. coli* feeding

C. elegans labeled with GCaMP3.0 in ASH neurons exposed to OP50 *E. coli* on TSA plates for 3 hours. Animals were exposed to aversive chemical 1 M glycerol starting at second 5 for 10 seconds.

Supplemental Video 2: Activity in *C. elegans* ASH neuron decreased after 3 hours of *S. aureus* infection

C. elegans labeled with GCaMP3.0 in ASH neurons exposed to SH1000 *S. aureus* on TSA + 10 µg/mL kanamycin plates for 3 hours. Animals were exposed to aversive chemical 1 M glycerol starting at second 5 for 10 seconds.

bioRxiv preprint doi: https://doi.org/10.1101/2025.05.01.651706; this version posted May 7, 2025. The copyright holder for this preprint (which was not certified by peer review) is the author/funder, who has granted bioRxiv a license to display the preprint in perpetuity. It is made available under aCC-BY 4.0 International license.

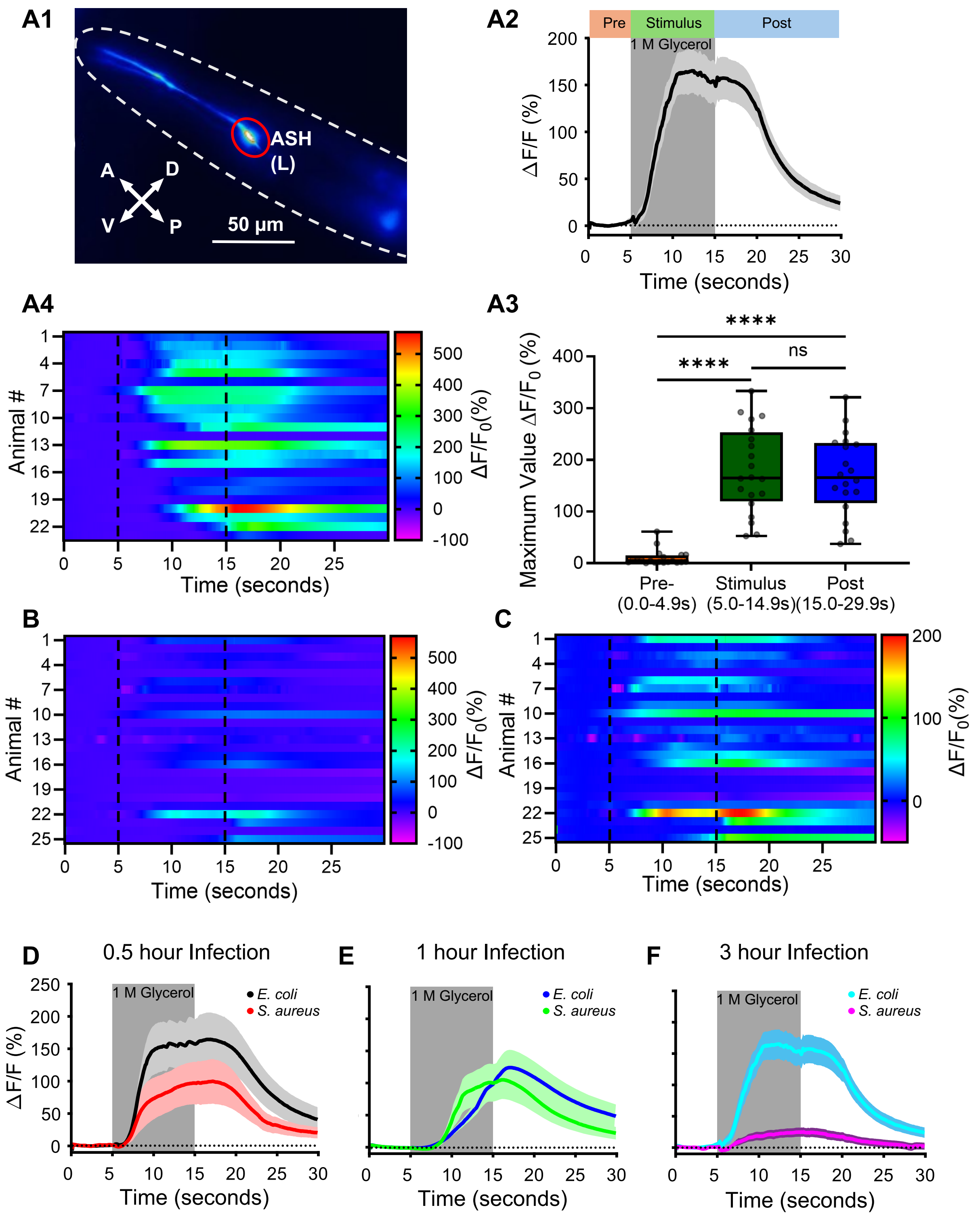

Supplemental Figure 1: *S. aureus* infection for 3 hours results in a significant decrease in ASH neurons physiology

bioRxiv preprint doi: https://doi.org/10.1101/2025.05.01.651706; this version posted May 7, 2025. The copyright holder for this preprint (which was not certified by peer review) is the author/funder, who has granted bioRxiv a license to display the preprint in perpetuity. It is made available under aCC-BY 4.0 International license.

A) Annotated workflow of calcium imaging on animals fed *E. coli* for 3 hours prior to imaging with 1 M glycerol. **A1)** Animals labeled with GCaMP3.0 in ASH neuron (promoter *sra-6*), soma (outlined in red) used as region of interest. **A2)** Normalized change in fluorescence of at least 10 animals (*E. coli* fed on TSA plates for 3 hours) are averaged and plotted with SEM, grey bar indicated application of stimulus, 1 M glycerol; **A3)** the maximum change in fluorescence values of each animal is plotted at the different time points of the stimulation, Pre (0-4.9 seconds) Stimulus (5-14.9 seconds), and Post (15.0-30.0 seconds), median plotted with minimum and maximum points. ($^{ns}p > 0.05$, $^{****}p < 0.0001$, Kruskal-Wallis test with Dunn's multiple comparisons). **A4)** heat map plotting response of every animal (1 row = 1 animal) across time of trial, arrow indicates how one point from the maximum peak graph can be mapped to heat map.

B) Heat map of all animals exposed to *S. aureus* for 3 hours, left same scale as **B)**

C) Heat map of all animals exposed to *S. aureus* for 3 hours adjusted scale to display variability in response

D-F) Percent change graphs ASH imaging of infected animals at **D)** 0.5, **E)** 1, **F)** 3 hours, stimulus of 1 M glycerol applied between 5-15 seconds.

bioRxiv preprint doi: https://doi.org/10.1101/2025.05.01.651706; this version posted May 7, 2025. The copyright holder for this preprint (which was not certified by peer review) is the author/funder, who has granted bioRxiv a license to display the preprint in perpetuity. It is made available under aCC-BY 4.0 International license.

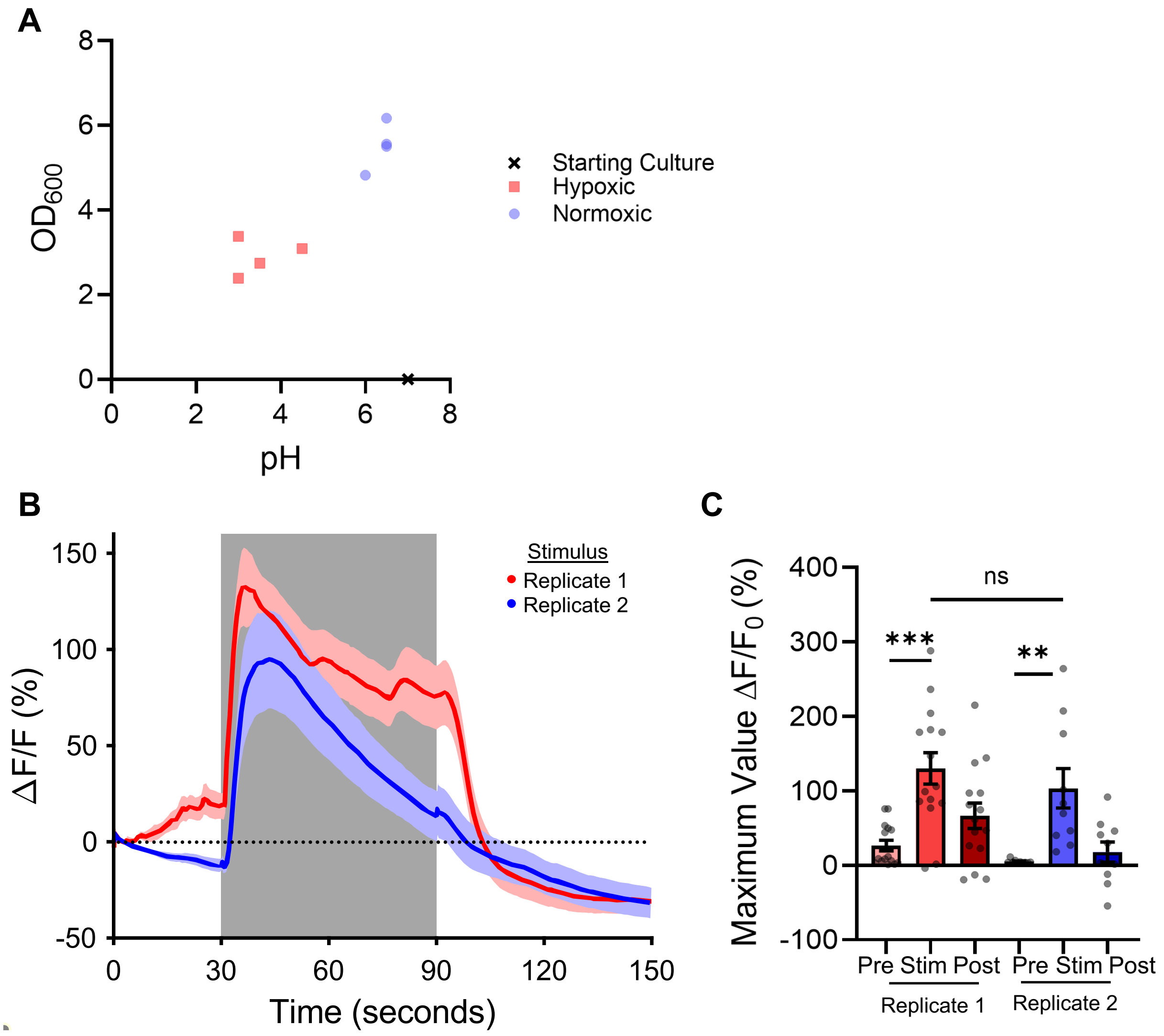

Supplemental Figure 2: ASH activity is suppressed by stationary phase *S. aureus*

A) OD_{600} and pH of 5 mL *S. aureus* cultures grown under normoxic or hypoxic conditions.

B) Response of ASH neurons to 1 M glycerol in TSB solvent, with TSB pre-exposure. Replicates performed twice throughout the course of experiments, a year apart. Trace is average response of n > 10 animals, SEM indicated with shaded region.

C) Maximum values of fluorescence intensity of neuron with background subtracted during times of pre-stimulus exposure (0-30 seconds), stimulus exposure (30-90

bioRxiv preprint doi: https://doi.org/10.1101/2025.05.01.651706; this version posted May 7, 2025. The copyright holder for this preprint (which was not certified by peer review) is the author/funder, who has granted bioRxiv a license to display the preprint in perpetuity. It is made available under aCC-BY 4.0 International license.

seconds), and post-stimulus exposure (90-150 seconds). Each data point is the value of an individual animal. (Replicate 1 (red) parametric paired t-test ***p = 0.0003, Replicate 2 (blue) non-parametric paired Wilcoxon test **p = 0.0020, between replicated parametric Student's unpaired two-tailed t-test ^{ns}p= 0.4381. All tests with Bonferroni correction, original *p ≤ 0.05, **p ≤ 0.01, ***p ≤ 0.001, corrected *p ≤ 0.025, **p ≤ 0.005, ***p ≤ 0.0005).

bioRxiv preprint doi: https://doi.org/10.1101/2025.05.01.651706; this version posted May 7, 2025. The copyright holder for this preprint (which was not certified by peer review) is the author/funder, who has granted bioRxiv a license to display the preprint in perpetuity. It is made available under aCC-BY 4.0 International license.

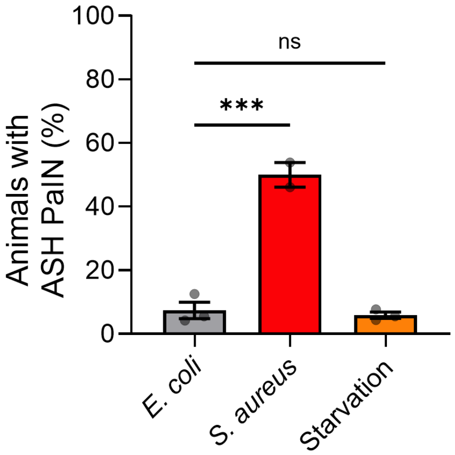

Supplemental Figure 3: *S. aureus* infection results in Pathogen Induced Neurodegeneration (PaIN)

Quantitative percent of ASH::GCaMP animals with ASH PaIN after fed *E. coli*, infected with *S. aureus*, or starved (TSA without bacteria) for 24 hours at 25 °C. Average ± SEM, three biological replicates; n = 15 - 25 animals per biological replicate. ($^{***}p = 0.0002$, $^{ns}p = 0.8804$; Unpaired two-tail t-test with Bonferroni correction, original values $^{ns}p \geq 0.05$, $^{***}p \leq 0.001$, corrected values $^{ns}p \geq 0.025$, $^{***}p \leq 0.0005$).
